# A soil-carrying lacewing larva in Early Cretaceous Lebanese amber

**DOI:** 10.1038/s41598-018-34870-1

**Published:** 2018-11-09

**Authors:** Ricardo Pérez-de la Fuente, Enrique Peñalver, Dany Azar, Michael S. Engel

**Affiliations:** 1 Oxford University Museum of Natural History, Parks Road, Oxford, OX1 3PW UK; 2 Instituto Geológico y Minero de España (Museo Geominero), C/Cirilo Amorós 42, 46004 Valencia, Spain; 3 State Key Laboratory of Palaeobiology and Stratigraphy, Nanjing Institute of Geology and Palaeontology, Chinese Academy of Sciences, Nanjing, 210008 People’s Republic of China; 4 Department of Biology, Faculty of Sciences II, Lebanese University, Fanar Matn, P.O. Box 26110217, Lebanon; 5 Division of Invertebrate Zoology, American Museum of Natural History, Central Park West at 79th Street, New York, 10024 USA; 6 Division of Entomology, Natural History Museum, and Department of Ecology & Evolutionary Biology, University of Kansas, 1501 Crestline Drive – Suite 140, Lawrence, Kansas 66045 USA

## Abstract

Diverse organisms protect and camouflage themselves using varied materials from their environment. This adaptation and associated behaviours (debris-carrying) are well known in modern green lacewing larvae (Neuroptera: Chrysopidae), mostly due to the widespread use of these immature insects in pest control. However, the evolutionary history of this successful strategy and related morphological adaptations in the lineage are still far from being understood. Here we describe a novel green lacewing larva, *Tyruschrysa melqart* gen. et sp. nov., from Early Cretaceous Lebanese amber, carrying a preserved debris packet composed by soil particles entangled among specialised setae of extremely elongate tubular tubercles. The new morphotype has features related to the debris-carrying habit that are unknown from extant or extinct green lacewings, namely a high number of tubular tubercle pairs on the abdomen and tubular tubercle setae with mushroom-shaped endings that acted as anchoring points for debris. The current finding expands the diversity of exogenous materials used by green lacewing larvae in deep time, and represents the earliest direct evidence of debris-carrying in the lineage described to date. The debris-carrying larval habit likely played a significant role during the initial phases of diversification of green lacewings.

## Introduction

Actively selecting, gathering, and carrying exogenous materials for mechanical protection and camouflage ‒debris-carrying, trash-carrying, or decoration‒ is a strategy that has evolved independently across a wide diversity of metazoans, including sea urchins, gastropods, and, above all, arthropods. In the latter, debris-carrying (excluding structure-building behaviour) is known in crustaceans (namely decorating crabs), arachnids (some oribatid mites and harvestmen, as well as sand-covering and walking mud spiders), and immature insects (assassin bugs, barklice, beetles, and neuropterans)^1–6^. Debris-carrying organisms have developed a series of behavioural and/or morphological adaptations aimed at fixing foreign elements to their bodies, such as sticky integumentary secretions or specialised (e.g., thorny or hooked) hair-like extensions of the integument (setae).

With about 1,400 known living species, Chrysopidae are the second most species-rich of the 20 living families currently recognised within Neuropterida^7,8^, and a well-studied insect group from an ecological standpoint due to the remarkable role that their predatory larvae have in biological pest control^9^. Chrysopids, and neuropterids in general, are a group that diversified during the Mesozoic and today shows a relict diversity and disparity^8^.

Two morphobehavioural types are distinguished among modern green lacewing larvae: the debris-carrying forms and the so-called “naked” ones. Naked forms do not construct a packet and tend to rely on swiftness and ambushing when hunting. On the contrary, debris-carrying forms use stealth and crypsis to capture their prey. Debris-carrying chrysopid larvae can be selective towards the collection of one or more materials for the construction of the debris packet. These materials may include sternorrhynchan waxy flocculence, arthropod exoskeletons, fragments of dried leaves, wood, lichens, mosses, trichomes, snail shells, silk, particles of sand or soil, frass (excrement of herbivorous insects), or the larvae’s own exuviae^10^. From all of these, only plant material (trichomes and vegetal debris) and exoskeletal fragments have been formerly reported as exogenous elements composing the debris packet from Cretaceous chrysopoid diversity^2,11,12^. On the other hand, intermediate stages between “pure” naked and debris-carrying forms exist whereby larvae show occasional debris-carrying or construct debris packets with loosely attached particles^10^. Debris-carrying larvae allocate the packet elements on their backs through a series of stereotyped movements arching the head backward while bowing the thorax and abdomen forward, followed by occasional peristaltic body movements to reallocate said packet elements^13,14^. In any case, both debris-carrying and naked forms are known to occur in two of the three subfamilies of Chrysopidae, i.e., Nothochrysinae and Chrysopinae (the latter subdivided into four tribes, Ankylopterygini, Belonopterygini, Chrysopini, and Leucochrysini). Only a debris-carrying mode of life is known among larvae of the remaining subfamily, Apochrysinae, but the larvae of only three species have been described^10,15^. Although it is clear that carrying debris is an ancient trait that has appeared or been lost multiple times during the evolution of larval chrysopoids, it is yet unclear whether it was the ancestral condition of the lineage^10^.

**Figure 1:**
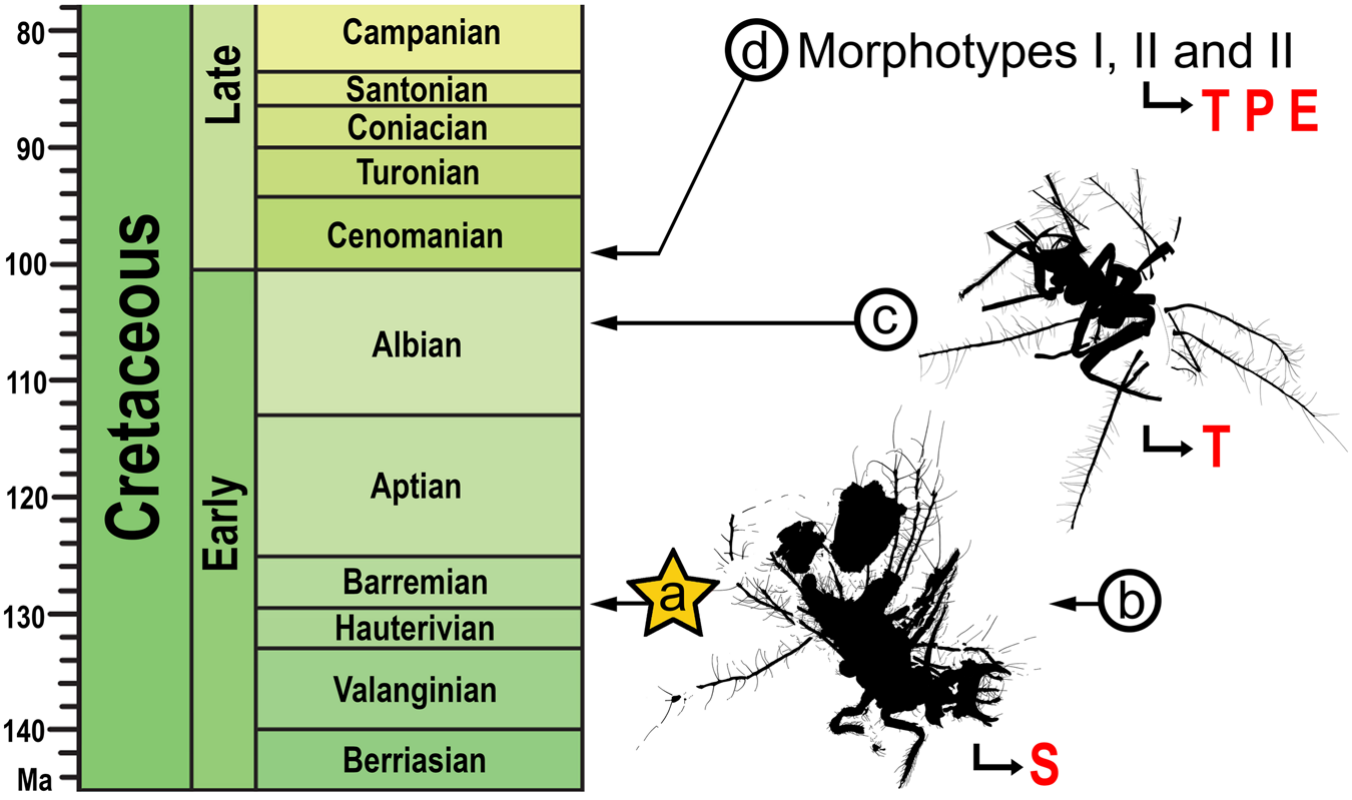
Cretaceous debris-carrying chrysopoid larvae known (bearing tubular tubercles) and materials composing their packet of debris, if present. (**a**) *Tyruschrysa melqart* gen. et sp. nov. (second or third instar, Lebanese amber) (this report). (**b**) New genus and species not associated to packet remains (first instar, Lebanese amber), to be described elsewhere. (**c**) *Hallucinochrysa diogenesi* (second or third instar, Spanish amber). (**d**) Three morphotypes based on 12 specimens, briefly characterised but unnamed or undescribed (Burmese amber). Drawings not to the same scale. Debris packet materials associated to the fossil larvae (red letters): T ‒ trichomes, P ‒ other plant material, E ‒ exoskeletons, S ‒ soil particles.

Fossil green lacewing immatures are scarce and only known from amber^2^. In Cretaceous amber, only one larval species of the debris-carrying morphotype ‒ showing extremely elongate tubular tubercles ( = TTs) forming a dorsal basket‒ has been previously described and named, the late instar *Hallucinochrysa diogenesi* from late Albian El Soplao amber (Spain)^2,11^. A new genus and species of neonate, also from Lebanese amber, will be described elsewhere (Pérez-de la Fuente *et al*., *submitted*) (Fig. 1). Additionally, three debris-carrying larval morphotypes recognised from 12 Burmese amber specimens were reported and briefly characterised^12^. Further diversity of Cretaceous chrysopoid larvae but not of the debris-carrying morphotype have been described ‒ an unnamed neonate clutching at the egg from where it hatched in Canadian amber, an extremely long-legged larva interpreted as a spider hunter in Burmese amber, and a larva mimicking liverworts also from Burmese amber^16–18^.

Here we describe a novel-looking chrysopoid larva with peculiar morphological adaptations for debris-carrying that is associated to debris packet elements interpreted as soil particles. The fossil is preserved in Early Cretaceous (Early Barremian, ca. 130 Ma) Lebanese amber, the oldest amber providing abundant biological inclusions^19^. The specimen, representing the oldest chrysopoid larva carrying a debris packet to be described, was previously figured and regarded as a new debris-carrying morphotype, but its debris packet was overlooked and the specimen was not named or described in detail^12^.

## Results

**Systematic Palaeontology**

**Order Neuroptera Linnaeus, 1758**

**Superfamily Chrysopoidea Schneider, 1851**

**Family** incertae sedis

**Genus** ***Tyruschrysa*** **gen. nov.**

LSID, urn:lsid:zoobank.org:act:57DE496F-8EB7-4CEB-81F8-0CE90136D42D.

**Type species**. *Tyruschrysa melqart* sp. nov.

LSID, urn:lsid:zoobank.org:act:B7C5A783-EBF8-4170-B996-E27C2A9CC2E8.

### Diagnosis (second or third instar larva)

Jaws short and wide, shorter than cephalic capsule. Antenna very short, shorter than jaws, with flagellum slightly expanding apically and lacking a terminal bristle. Prothorax lacking tubular tubercles (TTs); mesothorax with lateral and laterodorsal extremely elongate TT pairs, subequally developed, metathorax also with lateral and laterodorsal TT pairs, but laterodorsal pairs much more reduced. Abdominal segments (=A) 2‒6 with pairs of lateral TTs. A1‒A7 with pairs of laterodorsal TTs, thinner and shorter than lateral pairs. TT setae with mushroom-shaped endings. Abdomen with three sets of pairs of ventral tubercles bearing a few unspecialised setae: A2‒A8 with hemispherical lateroventral pairs of tubercles and smaller pairs of tubercles in a more submedial position ventrally (=submedioventral). A2‒A7 with pairs of weakly-developed tubercles between lateral TTs and lateroventral tubercles (=subpleural tubercles). Pretarsal claws without basal expansions.

### Etymology

From the Latin *Tyrus* (=Tyre), the ancient Phoenician city on the southern coast of Lebanon, after the allegorical resemblance of the series of tubular tubercles of the holotype from the type species and the allegedly Roman, colonnaded agora at the *Al Mina* excavation site, and *chrysa* (Greek, meaning “golden”), a traditional ending for chrysopoid genus-group names (gender: feminine).

***Tyruschrysa melqart*** **sp. nov**.

Figure 2‒5

2016 “Morphotype CIV, naked” Wang *et al*., fig. S1.F

**Figure 2:**
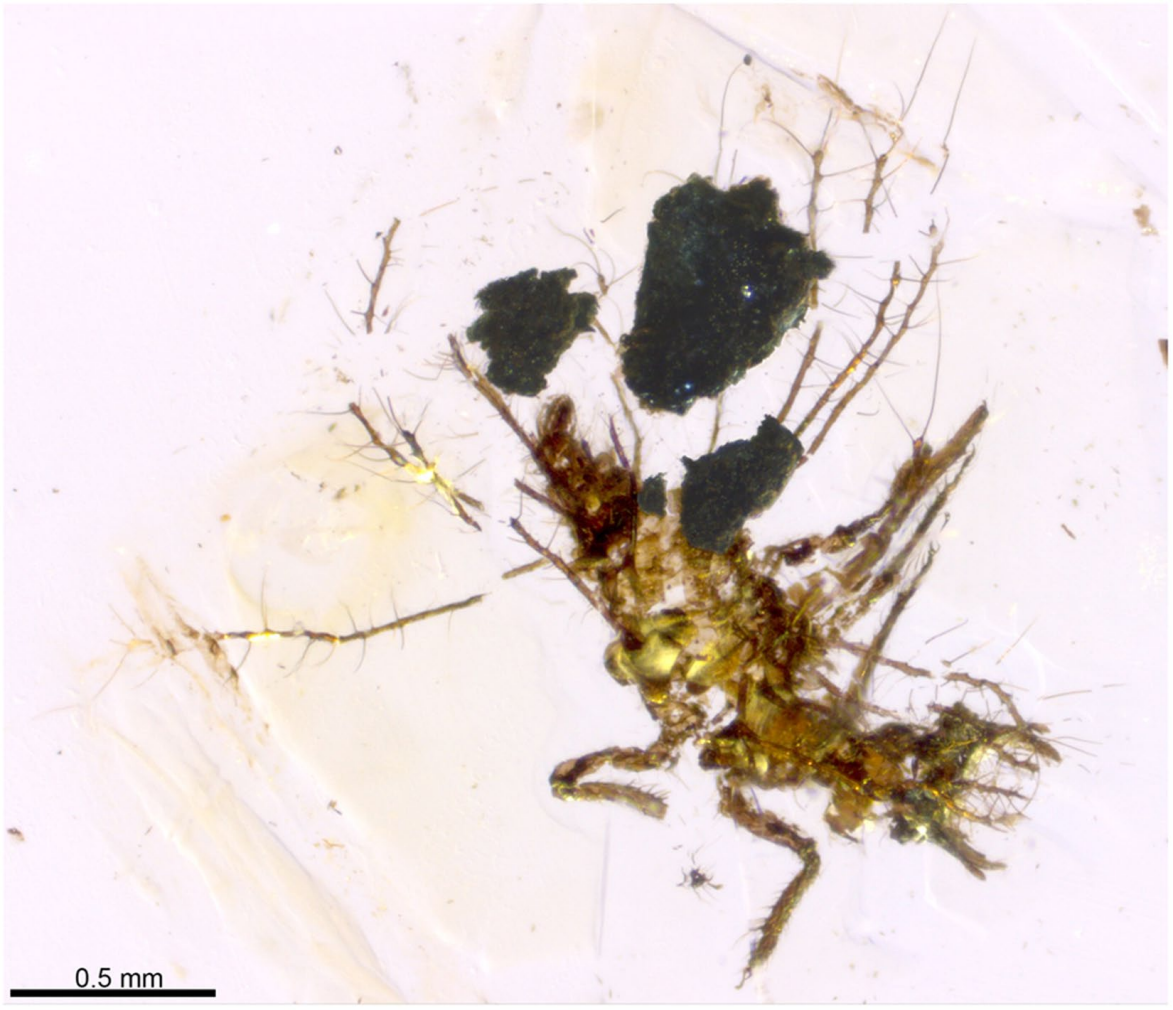
Dorsal habitus of *Tyruschrysa melqart* gen. et sp. nov., holotype TAR-96. The image is a composition of two images, one mostly using backlight to lit the larval structures and the second lighting the surface of the debris packet particles.

**Figure 3:**
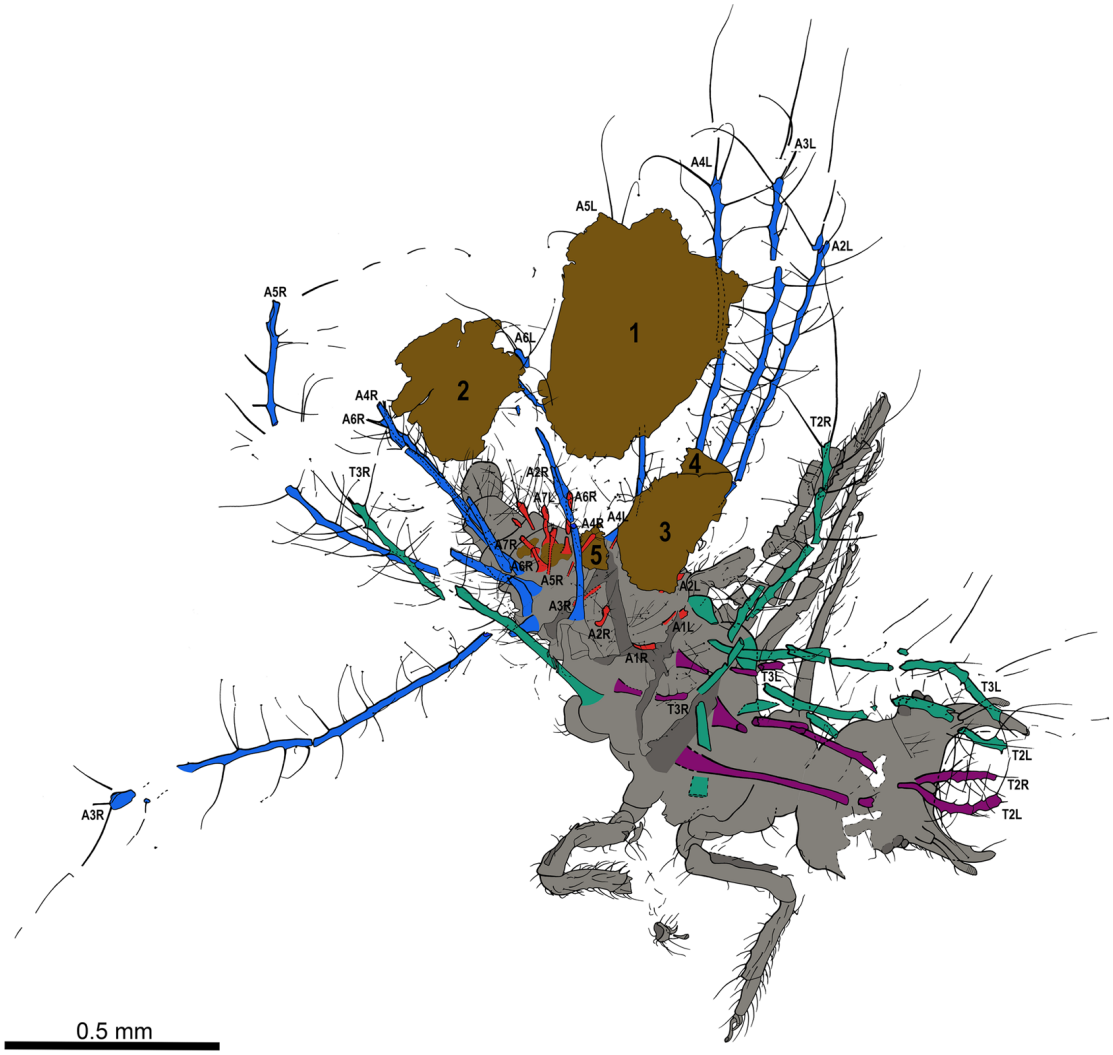
Camera lucida drawing of *Tyruschrysa melqart* gen. et sp. nov., holotype TAR-96. Tubular tubercles (TT) have been coloured: thoracic lateral TTs in green, thoracic laterodorsal TTs in purple, abdominal lateral TTs in blue, and abdominal laterodorsal TTs in red. The five particles composing the preserved debris packet (numbered) are shown in brown. Abbreviations: A – Abdominal TT, L – Left, R – Right, T2 – Mesothoracic TT, T3 – Metathoracic TT.

### Type material

Holotype TAR-96, an almost complete specimen carrying a preserved packet composed by soil particles. The specimen is deposited in the Natural History Museum of the Lebanese University, Faculty of Sciences II (Azar Collection), Fanar. The specimen is almost complete, only lacking the distoventral part of the cephalic capsule (including labial palps and the distalmost part of the maxillae). Preservation is relatively good but the specimen shows microfracturing and displacement in multiple points, likely resulting from preparation.

### Diagnosis

As for the genus (above).

### Description

**Figure 4:**
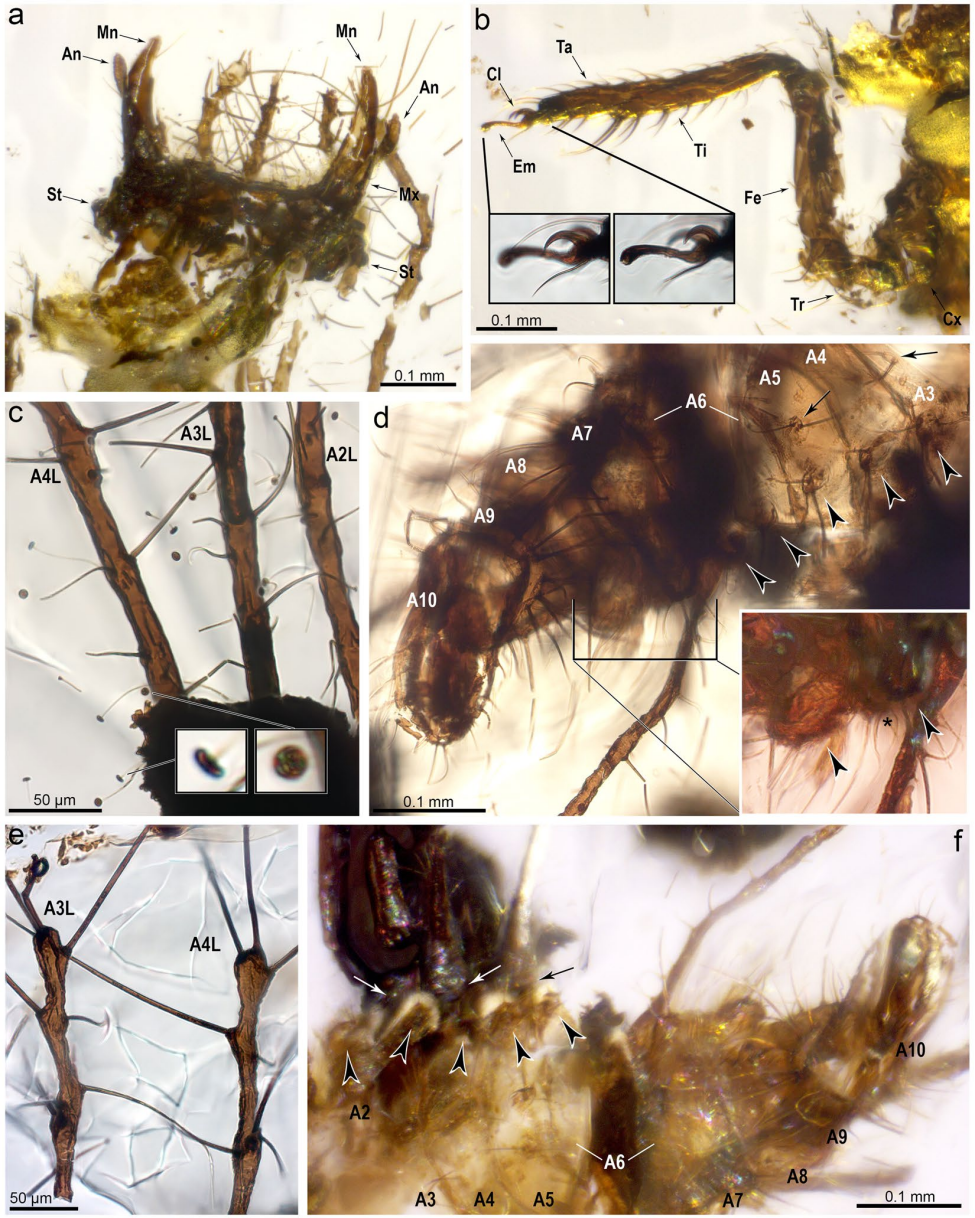
Morphological details of *Tyruschrysa melqart* gen. et sp. nov., holotype TAR-96. (**a**) Ventral view of head (fractured transversally). (**b**) Right foreleg. Insets show the shape of the two pretarsal claws. (**c**) Basal third portion of lateral left abdominal segments ( =A) 2‒4 TTs, showing setae and their mushroom-shaped ending. Insets show two enlarged setal endings (in lateral and ventral view, respectively). (**d**) Abdomen in ventral view, showing submedioventral (arrows) and lateroventral tubercles (arrowheads). Abdominal segments have been numbered. Inset shows lateroventral tubercles on A7 and A8 (arrowheads), as well as the subpleural tubercle on A7 (asterisk). (**e**) Distal portions of left A3 and A4 TTs. (**f**) Abdomen in ventral view, showing subpleural tubercles (arrowheads). Base of insertions of the lateral TTs are marked with arrows. Abbreviations: An ‒ Antenna, Cl ‒ Pretarsal claw, Cx ‒ Coxa, Em ‒ Empodium, Fe ‒ Femur, Mn ‒ Mandible, Mx ‒ Maxilla, St ‒ Stemmata, Ta ‒ Tarsus, Ti ‒ Tibia, Tr ‒ Trochanter.

*Second or third instar larva* (all measurements are in millimetres). Body campodeiform and gibbous. Body length ca. 1.4 from the head (excluding the jaws) to the apex of abdomen. Cephalic capsule subsemicircular in shape, ca. 0.24 long, 0.38 wide. Mandibles very short, 0.19 long, 0.05 wide at their base (ratio W/L = 0.26), rather stout, barely recurved; apices with a few sensilla, serrated inner margins present. Coupling structures at jaws absent. Maxillae only preserved basally and labial palps not preserved. Antennae very short, 0.17 long; scape cylindrical (0.04 long, 0.04 wide); pedicel 0.07 long; flagellum thick and short, 0.06 long, slightly expanding apically (not tapering), lacking terminal seta (Fig. 4a). Cranial sockets holding base of antennae absent. Ocular tubercles not prominent, with at least five stemmata although exact number not discernible. Several cephalic setae present, short; chaetotaxy not apparent but at least six very short setae emerging from the lateral sides of head, curving anteriorly, 0.04 long. No apparent colour pattern nor epicranial marks preserved.

Cervix subconical, rather elongate. Prothorax narrower than other thoracic segments, lacking setigerous tubular tubercles (=TTs). Mesothorax with lateral and laterodorsal pairs of elongate TTs, equally developed, anteriorly directed, about 0.70 long. Metathorax with lateral and laterodorsal pairs of elongate TTs, laterodorsal pairs much more reduced (0.20 long) than lateral ones (0.75 long). Setigerous TTs extremely elongate to moderately elongate, present on thorax and abdomen, with a rather constant thickness along their length (not becoming conspicuously thinner towards the apices), 0.01‒0.02 wide. Thoracic and abdominal TTs with a similar thickness. TT with setae along entire length, setae shorter basally (setae of abdominal TTs about 0.05 long basally and up to 0.40 long apically). TT setae emerging from tuberculated bases that are more conspicuous towards TT apex, smooth (without serrations), tapering apically, with mushroom-shaped endings (Fig. 4c). Some TT setae with less developed endings, almost knob-like, more common in distal areas of TTs. Body setae not emerging from TTs filiform (without expanded endings), both dorsally and ventrally. Chalazae not conspicuous. Insertion of thoracic TTs not particularly broad. Legs not particularly large and robust in relation to body, with scattered setae; prothoracic leg 0.73 long (coxa 0.09 long, trochanter 0.10 long, femur 0.23 long, tibia 0.20 long, tarsus 0.12 long); mesothoracic leg 0.76 long (coxa 0.11 long, trochanter 0.10 long, femur 0.23 long, tibia 0.21 long, tarsus 0.11 long); metathoracic leg about 1.13 long (coxa about 0.10 long, trochanter 0.18 long, femur 0.37 long, tibia 0.36 long, tarsus 0.12 long), about 2/3 the length of prothoracic leg. Trochanters without a conspicuous external, rounded process but bearing an especially elongate seta ventrodistally. Each tibia with a ventral row of three pairs of thickened setae, increasing in length and thickness towards apex (Fig. 4b); most apical pair of setae 0.08 long (on prothoracic leg). Tarsi with fused tarsomeres. Pretarsal claws sharp, recurving inwards, ca. 0.03 long, without laminar basal expansions (Fig. 4b). Trumpet-shaped empodia well developed (0.07 long on prothoracic leg). Two long, setae-like pulvilli present between claws, 0.04 long (on prothoracic leg).

Abdomen with two sets of dorsal TTs bearing setae with mushroom-shaped endings: five lateral TT pairs in abdominal segments (=A) 2‒6, and seven shorter laterodorsal TT pairs, about 0.20, in A1‒A7. Lateral TT pairs in A1‒A3 ~0.80‒0.85 long, A4 ~0.65 long, and A5 ~0.45 long. Abdomen bearing three sets of pairs of ventral setose tubercles: (1) weakly-developed pairs of tubercles in submedial position on A2‒A8 (=submedioventral tubercles), bearing two setae each; (2) hemispherical pairs of tubercles in lateroventral position on A2‒A8, each bearing three setae; and (3) weakly-developed pairs of tubercles between lateroventral tubercles and lateral TTs on A2‒A7, bearing a few setae each (=subpleural tubercles) (Fig. 4d). Lateroventral pair of tubercles in A7 especially well-developed and slightly projected ventrad, papilliform. Pair of ventral tubercles in A8 particularly broadened, papilliform, likely fusion of lateroventral tubercle and subpleural tubercle from same abdominal segment. A9 and A10 subcylindrical, latter (apex) relatively large. A9 with a ventrodistal fan of 7‒8 remarkably elongate and thick setae (Fig. 4d,f).

### Age and locality

Early Cretaceous, Early Barremian^20^, Bouarij, Caza Zahleh, Mohafazat El-Beqaa, Central Lebanon^19^.

### Etymology

The specific epithet is after the most accepted transcription of the Phoenician god “*Milk-Qart*” (originally meaning “king of the city”, also spelled Melquart, Melkart, or Melkarth), tutelary deity of the city of Tyre and a mighty hunter that was identified with the divine hero Heracles by the Greek. The name, which is treated as a noun in apposition, refers to the powerful appearance that carrying large pieces of debris gives to the holotype and the predatory habits of green lacewing larvae.

## Remarks

**Figure 5:**
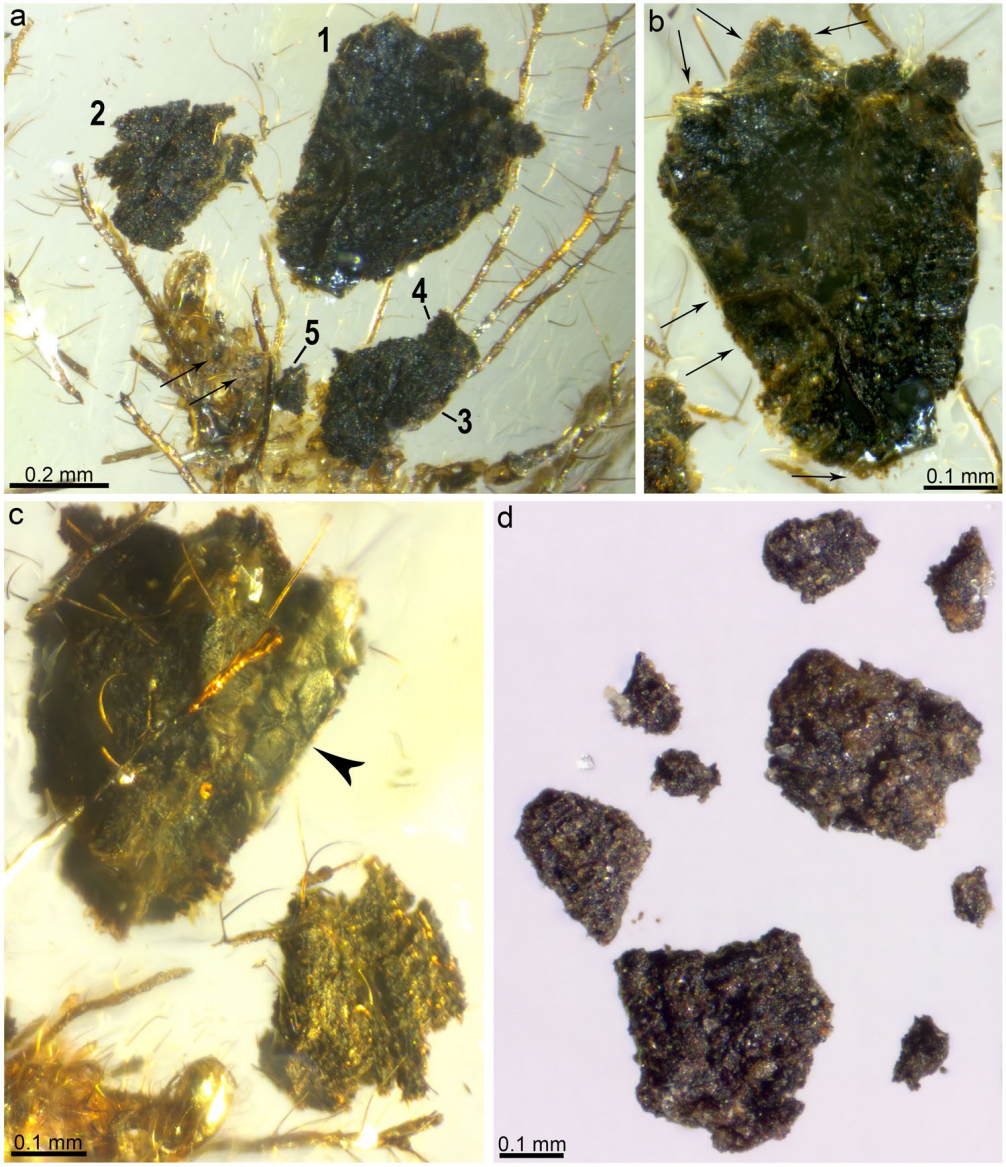
Particles from the preserved packet of debris of *Tyruschrysa melqart* gen. et sp. nov., holotype TAR-96. (**a**) Complete view of the five preserved packet particles, numbered. Arrows point to a disaggregated cloud of particles attached to the distalmost laterodorsal TTs on the abdomen. (**b**) Detail of the largest particle (numbered 1 in **a**). Arrows point to translucent films. (**c**) The two largest particles (numbered 1 and 2 in **a**) viewed from the opposite angle leaning on lateral tubular tubercles from the abdomen. Arrowhead points to a cracked surface. (**d**) Sample of freshly collected soil particles, imaged dry (no immersion) for comparison.

Five black, opaque, irregularly-shaped particles are in contact with the tubular tubercles and the setae with mushroom-shaped endings emerging from them. These dense particles have different sizes: particle #1 is 0.43 to 0.62 mm, #2 is 0.19 to 0.33 mm, #3 is 0.26 to 0.31 mm, #4 is 0.09 to 0.13 mm, and #5 is 0.07 to 0.10 mm (Fig. 5a). Although most surfaces of the particles are granulose and porous, one surface is relatively flat and shows distinct cracking (Fig. 5c). Most surfaces are coated by lighter (brownish to greenish), translucent films (Fig. 5b). Additionally, cloudy material, likely disaggregated from the dense particles, is present attached to the last laterodorsal tubercles of the abdomen (Fig. 5a).

The specimen corresponds to a late instar (i.e., second or third instar) due to: (1) the low relative size proportion of the legs and the head in relation to the body, (2) the high degree of setation and tubercle development, and (3) the humped body, all of the above when compared to extant green lacewing first instar larvae and the neonate species to be described elsewhere from Lebanese amber (Pérez-de la Fuente *et al*., *submitted*). *Tyruschrysa melqart* gen. et sp. nov. could have been the larval form of one of the multiple chrysopoid lineages described from the Cretaceous based on adult material^21^.

The new species was previously figured in the supplementary information of Wang *et al*.^12^. Although those authors considered the specimen as belonging to the debris-carrying morphotype IV (in which four specimens, all from Lebanese amber, were included), TAR-96 was treated as “naked”. Therefore, the particles present in the sample (Fig. 5) were overlooked as debris packet components, likely a result of not having had the chance to examine the specimen. Moreover, it is worthy to note that, contrary to the brief descriptive note offered by Wang *et al*.^12^ on morphotype IV, i.e., “The body is flat, with length about 4 mm. The head is approximately 1 mm long. The jaws are shorter than the head. The tubular tubercles are 3‒4 mm long”, the body of *T. melqart* is clearly gibbous, and the lengths of body, cephalic capsule, and TTs are less than half the lengths originally provided^12^.

The previously described chrysopoid larva *H. diogenesi* shares with the new species a body dorsum with pairs of setigerous, extremely elongate tubercles (i.e., tubular tubercles), exceeding the body width; at least the mesothoracic segment with two pairs of tubular tubercles (lateral and laterodorsal pairs); and the abdomen with at least one pair of smaller tubular tubercles in laterodorsal position. The figured debris-carrying larvae from Burmese amber reported and figured by Wang *et al*.^12^ (at least morphotypes CI and CII) appear to share these characters as well, although detailed descriptions of these specimens are necessary. We refrain from creating further taxonomic subdivisions pending further descriptions and discoveries that will help expand the disparity of characters among fossil debris-carrying chrysopoids and test their stability. *Tyruschrysa melqart* differs from *H. diogenesi* in the shape of the head (subsemicircular vs. banana-shaped), the mandibles (shorter than the head, stout vs. longer and stylised), and the antennae (shorter than the mandibles vs. longer), the morphology of the TT setal endings (mushroom-shaped in the new species vs. trumpet-shaped), the relative size of the legs (smaller in the new species), and the lack of pretarsal claw basal expansions, among other characters. It is noteworthy that the blunt and short jaws and the lack of a terminal elongate seta (bristle) on the antennae are unusual characters among modern chrysopids. Indeed, although in extant Chrysopidae the jaws are stylet- and scythe-like, and longer than the cephalic capsule in most of the species, a few of them have blunt jaws shorter than the cephalic capsule, as in the new fossil species, such as some Belonopterygini^22,23^. Likewise, most extant chrysopids have an elongate seta on the antennal flagellum, but its absence is considered a synapomorphy of the third larval instars within the Nothochrysinae^24^.

## Discussion

The five particles preserved together with *Tyruschrysa melqart* gen. et sp. nov. are confidently regarded as bits of debris gathered by the own larva. The morphological characteristics of these particles are consistent with them being soil particles containing decaying organic matter such as bark as well as microbial/fungal growths (Fig. 5d). Modern ecological analogues of *T. melqart* have been described among extant debris-carrying chrysopids: *Italochrysa insignis* gathers soil and dried plant material to construct a highly compacted packet. The resulting dome-like packet confers protection against the attacks of arboreal ants as the chrysopid larvae feed on ant larvae and pupae in the ant nests^23^.

In general terms, whereas debris-carrying larvae tend to have globose, gibbous bodies and well-developed (up to digitiform) tubercles bearing abundant, relatively long, textured setae with curved endings, naked larvae have fusiform, flat bodies and weakly-developed (up to hemispherical) tubercles bearing sparse, short, smooth setae with acute or blunt endings^10^. *Tyruschrysa melqart* was clearly a debris-carrying morphotype, but both its tuberculation pattern and tubular tubercle setae are peculiar, unknown from extant chrysopid larvae and their extinct relatives described.

**Figure 6:**
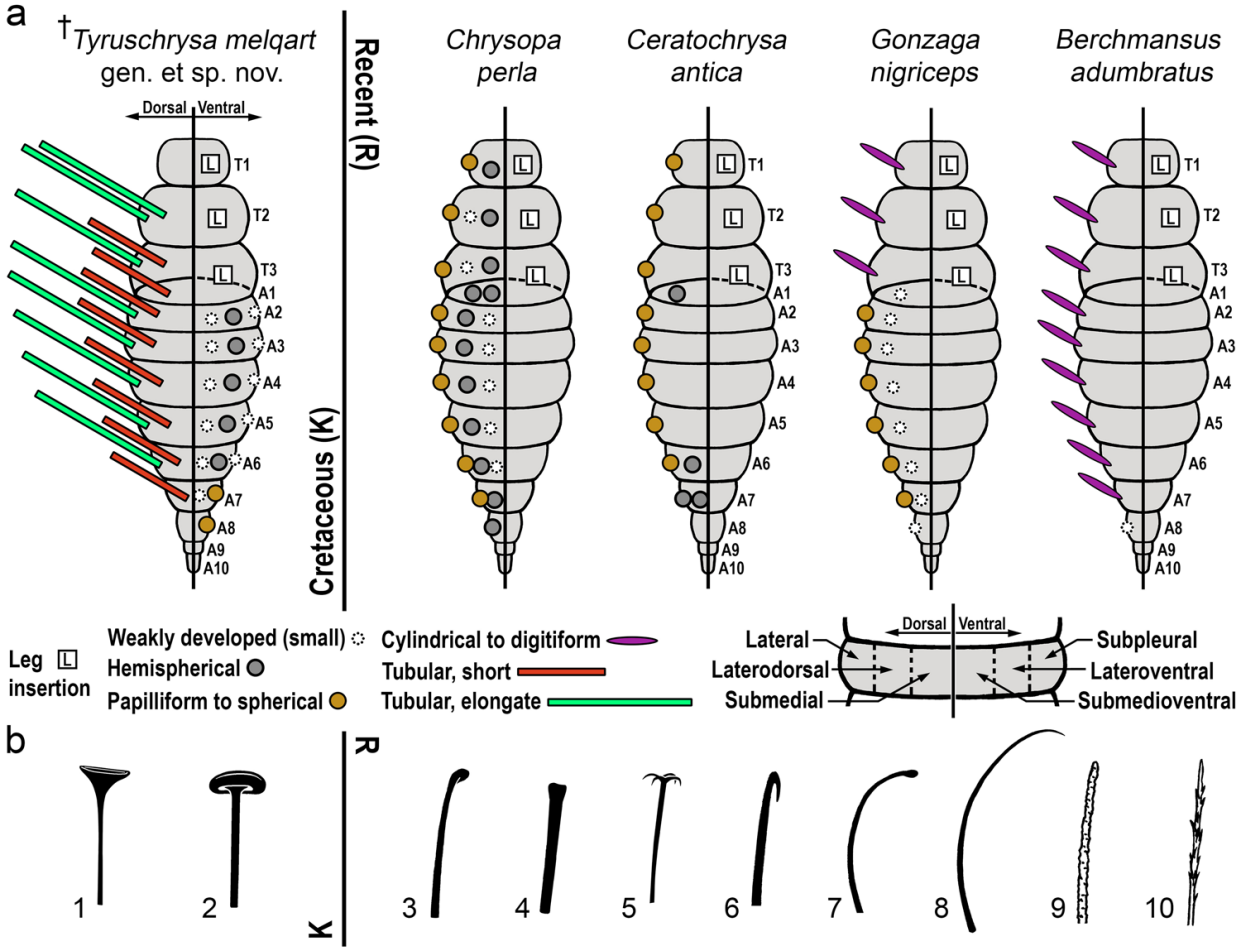
Tuberculation pattern and setal specializations present in Cretaceous (left) and Recent (right) chrysopoids (not to the same scale). Data extracted from^26,29,31,38,39,52^. (**a**) Tubercle arrangement and development in the new species and selected extant chrysopid representatives (third instars, all known to be debris-carrying in a lesser or greater degree but *B. adumbratus*, which mode of life is unknown). Types of tubercles based on their morphology and anatomical position in the segment (dorsally and ventrally) are provided in the legend. Larval bodies shown are idealised. The tuberculation pattern is unknown in *Hallucinochrysa diogenesi* due to preservation and therefore it has not been included. Note that although ventral tuberculation has not been depicted in the Recent morphotypes due to the lack of description of these structures in the corresponding literature, this character might have been overlooked at times, at least for some chrysopinines, and should therefore be treated with caution (see text). (**b**) Types of specialised setae found in debris-carrying (including occasional forms) chrysopoid larvae. 1. Trumpet-shaped ending, 2. Mushroom-shaped ending, 3. Spatulate ending, 4. Knobbed ending, 5. Multipronged ending, 6. Hook-like ending, 7. Curled (uncinate) seta with spatulate ending, 8. Broadly curved seta, 9. Denticulate seta, 10. Thorny seta. Setae 1‒8 are smooth, 9 and 10 are textured.

The number and, above all, the degree of development of pairs of setose tubercles in green lacewing larvae are variable and usually correlated with the naked or debris-carrying mode of life. Setose tubercles are usually lateral in position, but additional pairs can be developed laterodorsally and even submedially^10,25^. Lateral tubercles are better developed than laterodorsal ones, and the latter tend to be better developed than the submedial tubercles, when present. With the exception of some naked forms (e.g., *Brinckochrysa*, *Hypochrysa*, *Pimachrysa*) where tubercles are absent or vestigial^26,27^, extant chrysopid larvae show distinct pairs of setose tubercles on the dorsum of the three thoracic segments and of a variable number of abdominal segments. In any case, setose tubercles as dramatically elongate (=tubular) as in the Cretaceous chrysopoids are absent from modern chrysopid larvae, as their degree of development only ranges from weakly-developed (“small”), to hemispherical, papilliform, spherical, cylindrical, and digitiform. The longest digitiform tubercles known in extant chrysopid larvae are not, in any case, longer than the width of the larval body, and are laterally-placed on the dorsum of the thoracic segments from the debris-carrying species of Leucochrysini^28–31^. The third instar of a species classified in the latter, *Berchmansus adumbratus*, is highly unusual in that not only the thoracic but also the abdominal lateral tubercles are digitiform, giving a superficial resemblance to the extinct chrysopoids bearing tubular tubercles^31^ (Fig. 6a). It is interesting to note that the condition of possessing digitiform lateral tubercles is commonly present in the larvae of owlflies (Ascalaphidae) and split-footed lacewings (Nymphidae) (named scoli in these groups), the latter even possessing additional scoli placed laterodorsally^32–36^. Moreover, the first thoracic segment of extant debris-carrying chrysopid larvae (all instars) bears, at least, a pair of lateral tubercles. This is a major difference contrasting with the lack of tubercles on the first thoracic segment in *Tyruschrysa* and also some undescribed forms from Burmese amber^12^. This absence could be related to the extreme elongation of the lateral tubercles having imposed mechanical restrictions during the packet loading phase. Apart from pairs of lateral tubercles, pairs of laterodorsal tubercles can occur in all thoracic segments and most abdominal ones (excluding the distalmost segments), such as in some naked or occasionally debris-carrying species of *Chrysopa*^26^ (Fig. 6a). However, laterodorsal tubercles are more commonly only present on the abdomen: these can be distinct on most segments, i.e., A1‒A7 or A1‒A8, both in third instars of debris-carrying forms such as species in the genera *Apochrysa*, *Gonzaga*, *Leucochrysa*, and *Santocellus*^15,28–30^ (Fig. 6a) or naked/light debris-carrying Chrysopini such as *Peyerimhoffina* and *Plesiochrysa*^26,37^. Conversely, laterodorsal tubercles can be restricted to particular abdominal segments in debris-carrying forms, namely on A6 and A7 (*Chrysopodes*, *Suarius*), A5‒A7 (*Pseudomallada*), or A1, A6, and A7 (*Ceraeochrysa*, *Ceratochrysa*)^26,38–40^ (Fig. 6a). In all of these cases, first instars tend to show a higher degree of tuberculation, with a higher number of laterodorsal and submedial tubercles than second or third instars. On the other hand, the presence of three sets of pairs of hemispherical to weakly-developed tubercles in ventral position on the abdomen (named herein subpleural, lateroventral, and submedioventral pairs, Fig. 6a) each bearing a few (at least two) setae is a condition that, to our knowledge, has not been formally described from any extant or extinct green lacewing larva. Nevertheless, the presence of ventral tubercles on the abdomen appears to have been neglected at times in modern taxa, at least in some chrysopinines. This is based on pictures of larvae in lateral view where alleged tubercles bearing setae in subpleural position are visible (*vide Chrysopa oculata* and an unconfirmed *Pseudomallada* sp.^10^), as well as on a brief reference to “protuberances ventrally and sublaterally”, each “bearing a long, curved bristle” in first instars and “with a few short bristles” in the third instar of *Pseudomallada flavifrons*^41^. Note that larvae within several *Chrysopodes* species show protuberances ventrally on the abdomen, below the lateral tubercles (in subpleural position)^38^, but these are minute and bear a single seta, therefore falling under the category of chalazae rather than true tubercles bearing more than one setae. Likewise, pairs of “chalazae ventral to lateral abdominal tubercles” were described in *Chrysoperla carnea*^42^. It is also important to note that setose tubercles or ventral abdominal protuberances of any kind are absent in the drawings/pictures from some first and third instar chrysopinines, such as in *Chrysopa* and *Kymachrysa* species, respectively^43,44^. Therefore, although the presence of three sets of pairs of true, well-developed ventral tubercles on the abdomen is a condition that could be exclusive of *T. melqart*, it would appear as if these structures currently have an ill-documented variability in modern chrysopid (namely chrysopinine) larvae that is worthy of reassessment. On the contrary, weakly defined tubercles (setiferous processes) bearing abundant setation are well known to occur ventrally on the abdomen from extant larvae belonging to other neuropteran families such as antlions (Myrmeleontidae) and spoon- and thread-winged lacewings (Nemopteridae)^45,46^. The ventral position on the abdomen of the tubercles, their low degree of development, and their setae lacking expanded endings (as in the dorsal TTs) suggests that these were not used by *T. melqart* to allocate debris, at least actively. One option is that these tubercles did not serve a particular function but that they were allometrically developed due to the general high degree of tuberculation shown by the larva.

The presence of smooth setae (not textured) with expanded endings, a character shared by *H. diogenesi* and *T. melqart* but unknown from the diversity of modern green lacewing larvae, exemplifies yet another type of adaptation for enhancing the debris-carrying capabilities of these setae. Setal endings are trumpet-shaped in *H*. *diogenesi*^2^ and relatively larger, mushroom-shaped in *T*. *melqart* (Fig. 6b), but both acted as anchoring points for the debris-packet elements. Interestingly, setae with such expanded endings (particularly resembling those of *H. diogenesi*) have been described from the extant larval diversity of nymphids^34^. Overall, larval neuropterans are characterised by a remarkable morphological diversity of their body setae. Specialised setae are also known from the larvae belonging to other neuropteran families, namely from myrmeleontiforms such as ascalaphids, nemopterids, nymphids, myrmeleontids, and psychopsids^32,35,45–48^, but also from other neuropterans such as hemerobiids and sisyrids^49,50^. Perhaps the most striking reported setal diversity is that of Ascalaphidae, in which greatly modified setae can range from goblet-shaped to scale-like or plumose^32^. Nonetheless, the diversity of tubercle setae in modern chrysopid larvae is quite comparable, and it includes both specialised endings and textured surfaces (Fig. 6b). These setal specialisations are usually aimed at increasing the retention of the packet elements in debris-carrying forms, although they can also be present in naked forms. Among these, expanded, blunt endings are the setal specialisations most similar to the trumpet-/mushroom-shaped setal endings described from fossil chrysopoid larvae. These include spatulate (=spoonbilled) setae, present in debris-carrying (e.g., *Chrysopidia*, *Cunctochrysa*, *Pseudomallada*, and *Suarius*) and naked (e.g., *Peyerimhoffina*) Chrysopini^26^; and knobbed (=clavate) setae (as in *Berchmansus* or *Anomalochrysa*, occasionally debris-carrying)^31,51^. Further specialisations encompass multipronged (=truncated) setal endings, known in debris-carrying *Vieira* species and also in naked forms within *Brinckochrysa*^26,52^. However, the most common setal specialisation associated with debris-carrying is hook-like endings, present in species of genera such as *Apochrysa*, *Ceraeochrysa*, *Gonzaga*, *Leucochrysa*, *Pseudomallada*, *Santocellus*, *Suarius*, or *Rexa*^26,28,29,40,53,54^. Flattened hook-like setal endings are known in *Chrysopodes*^38^. Hook-like setal endings can also be present in light debris-carriers (e.g., *Plesiochrysa*) or occasionally debris-carrying/naked forms as some Chrysopini (*Atlantochrysa*, *Chrysopa*, and *Meleoma*)^10,37,55,56^. These endings are sometimes difficult to distinguish from curled (recurved) setae, both broadly (e.g., *Chrysopa*) or narrowly as in *Nothochrysa* ( = uncinate setae)^26^. Curled setae sometimes have hook-like endings (e.g., *Chrysopa*), but these can also be spatulate (*Pseudomallada, Cunctochrysa*) or multipronged (*Vieira*) (see above). Furthermore, a different type of setal specialisation that tends to be associated to debris-carrying among modern chrysopids is the presence of textured surfaces, i.e., setae with more or less developed projections along their length. This includes setal projections that are, in order of increasing development, granular as present in *Kymachrysa*^44^, denticulate as in *Italochrysa*^26^, thorny/serrated as present in *Ceraeochrysa*, *Chrysopodes*, *Leucochrysa*, or *Suarius*^26,38,40,54^, and plumose as in *Ankylopteryx*^7^.

The holotype of *T. melqart* is carrying soil particles corresponding to an initially built debris packet or a partially dislodged one resulting from resin entrapment. These particles were selected, gathered and carried by the larva, and their presence on the specimen represent direct evidence that the debris-carrying strategy and associated behaviours have been present in larvae of the chrysopoid lineage for, at least, about 130 million years. The selective pressures that could explain why the larvae of these Cretaceous lineages of green lacewings possessed extremely elongate tubular tubercles are still unclear. It was previously hypothesised that such extremely elongate tubercles would have allowed the construction of a particularly thick debris packet, and therefore might have been adapted to protect from predators or parasitoids with elongate piercing structures such as proboscides or ovipositors^2^. In light of the fossils described so far, it would appear as if the debris-carrying condition in the chrysopoid lineage shifted from forms with extremely elongate (tubular) tubercles bearing setae smooth and with expanded endings to forms with much shorter tubercles but bearing setae typically textured or hooked-tipped/curved. Alternatively, these two groundplans (if distinct) could have coexisted. In any case, it is clear that more fossils are required, including detailed descriptions of the abundant chrysopoid larvae reported from Burmese amber^12^, in order to start assessing the relationships among the different stem-group larval chrysopids and to test evolutionary trends in the chrysopoid lineage. Although some of the characters from the new fossil are unknown from modern relatives, such as elongate lateral and laterodorsal tubercles on most abdominal segments, tubercle setae with expanded endings, and sets of pairs of tubercles bearing a few setae each in a ventral position on the abdomen (tentatively exclusive of the fossil pending for a reassessment of these structures in modern chrysopids), similar features are today found among other neuropteran larvae from the myrmeleontiform lineage, namely in myrmeleontids, nymphids, and/or ascalaphids. This suggests that these characters could potentially be plesiomorphic for *T. melqart*, being lost during the evolution of chrysopoids, or, more likely, independently evolved relative to those in the Myrmeleontiformia. Again, the description of more extinct diversity is required to test this and further hypotheses. It is clear that larval studies have proven useful to shed light on the species- and suprageneric-level relationships of Chrysopidae^10^. Gathering fossil evidence suggests that the debris-carrying habit of larvae played an important role for the diversification of green lacewings during the early evolutionary stages of the lineage. Fossil chrysopoid larvae will no doubt be paramount in unravelling the evolutionary history of this remarkable insect group and that of protective-camouflaging strategies as successful as the debris-carrying.

## Material and Methods

The studied specimen, TAR-96, was isolated within a small amber piece by removing the main part of the amber around it, and then prepared between two circular cover slips in Canada balsam medium^57^.

A Discovery.V12 Zeiss stereomicroscope, and two compound microscopes, an Olympus BX51 and a Zeiss AXIO, were used to study the specimen in the two views that the preparation permits. The specimen was illustrated using a drawing tube Olympus U-DA attached to the Olympus BX51 and photographed using an Axiocam 105 colour digital camera attached to both the stereomicroscope and the Zeiss AX10. The scanned camera lucida drawing of the specimen was coloured with Adobe Photoshop CC version 18.0. Microphotographs were stacked using the software Helicon Focus version 6.80.

Nomenclature used follows that of C. A. Tauber and co-workers^10^.

This published work and the associated nomenclatural acts have been registered in ZooBank, the proposed online registration system for the International Code of Zoological Nomenclature. The ZooBank LSIDs (Life Science Identifiers) can be resolved and the associated information viewed through any standard web browser by appending the LSID to the prefix “http://zoobank.org/”. The LSID for this publication is 97C1C42D-EC87-40DF-93F6-73C7AAF11546, and those of the associated nomenclatural acts are 57DE496F-8EB7-4CEB-81F8-0CE90136D42D (Tyruschrysa gen. nov.) and B7C5A783-EBF8-4170-B996-E27C2A9CC2E8 (T. melqart sp. nov.).
